# Neutrophil and Alveolar Macrophage-Mediated Innate Immune Control of *Legionella pneumophila* Lung Infection via TNF and ROS

**DOI:** 10.1371/journal.ppat.1005591

**Published:** 2016-04-22

**Authors:** Pascal Ziltener, Thomas Reinheckel, Annette Oxenius

**Affiliations:** 1 Institute of Microbiology, ETH Zürich, Zürich, Switzerland; 2 Institute for Molecular Medicine and Cell Research and Centre for Biological Signalling Studies, Albert-Ludwigs-University Freiburg, Freiburg, Germany; Purdue University, UNITED STATES

## Abstract

*Legionella pneumophila* is a facultative intracellular bacterium that lives in aquatic environments where it parasitizes amoeba. However, upon inhalation of contaminated aerosols it can infect and replicate in human alveolar macrophages, which can result in Legionnaires’ disease, a severe form of pneumonia. Upon experimental airway infection of mice, *L*. *pneumophila* is rapidly controlled by innate immune mechanisms. Here we identified, on a cell-type specific level, the key innate effector functions responsible for rapid control of infection. In addition to the well-characterized NLRC4-NAIP5 flagellin recognition pathway, tumor necrosis factor (TNF) and reactive oxygen species (ROS) are also essential for effective innate immune control of *L*. *pneumophila*. While ROS are essential for the bactericidal activity of neutrophils, alveolar macrophages (AM) rely on neutrophil and monocyte-derived TNF signaling via TNFR1 to restrict bacterial replication. This TNF-mediated antibacterial mechanism depends on the acidification of lysosomes and their fusion with *L*. *pneumophila* containing vacuoles (LCVs), as well as caspases with a minor contribution from cysteine-type cathepsins or calpains, and is independent of NLRC4, caspase-1, caspase-11 and NOX2. This study highlights the differential utilization of innate effector pathways to curtail intracellular bacterial replication in specific host cells upon *L*. *pneumophila* airway infection.

## Author Summary

*Legionella pneumophila* is a motile gram-negative bacterium found mainly in fresh water environments where it replicates in amoeba. It uses a molecular syringe to inject effector molecules into these predatory host cells, reprograming them to support *L*. *pneumophila* growth. Upon inhalation of contaminated aerosols, *L*. *pneumophila* uses the same approach to replicate in human alveolar macrophages, which can result in a severe pneumonia known as Legionnaires’ disease. However, *L*. *pneumophila* is normally controlled by the innate immune system, and the key mechanisms and cells involved in this immune response remain unclear. Here we show that tumor necrosis factor (TNF) and reactive oxygen species (ROS) play a dominant role in the clearance of *L*. *pneumophila* from the lung. Neutrophils kill *L*. *pneumophila* using ROS, while alveolar macrophages are activated by TNF produced by neutrophils and monocytes that are recruited to the lung. TNF-activated alveolar macrophages kill *L*. *pneumophila* by recruiting lysosomes and acidifying *L*. *pneumophila* containing vacuoles. Caspases other than caspase-1 and 11 are involved in this mechanism, with a minor contribution from cysteine-type cathepsins or calpains. This study deepens our understanding of the mechanisms by which TNF contributes to the control of intracellular pathogens, and highlights the key elements of the innate immune response to *L*. *pneumophila* lung infection.

## Introduction

*L*. *pneumophila* is a Gram-negative bacterium with global distribution in freshwater environments, where it replicates intracellularly mainly in amoebae [1–3]. *L*. *pneumophila* commonly causes community acquired and nosocomial pneumonia. Although it is normally controlled by the innate immune response, *L*. *pneumophila* has the potential to cause a severe pneumonia known as Legionnaires' disease with mortality rates of up to 30% if early bacterial replication is not controlled [4–6]. Infection occurs through inhalation of *L*. *pneumophila* contaminated aerosols, mostly generated by manmade technologies such as cooling towers, air conditioners or even car windshield wipers [7–9]. In the lung *L*. *pneumophila* initially exclusively infects alveolar macrophages (AM), using a type IV secretion system (T4SS) to inject over 300 effector proteins into the cytosol [7,10–12]. These effectors block phagosomal maturation and fusion with lysosomes, thus preventing *L*. *pneumophila* degradation, and promoting the establishment of a Legionella containing vacuole (LCV), the intracellular niche in which *L*. *pneumophila* replicates [13–16].

Though critical for *L*. *pneumophila* replication, the T4SS also potently induces the innate immune response by several mechanisms (reviewed in [17]). AM sense the action of the T4SS and respond by secreting IL-1α, inducing the secretion of chemokines by airway epithelial cells (AECs), resulting in the rapid recruitment of neutrophils and monocytes to the lung [10,18,19]. Neutrophils are known to be critical for the clearance of *L*. *pneumophila* lung infection, as evidenced by neutrophil depletion studies [18,20,21], *in vivo* blockade of CXCR2 [22] and studies examining the role of IL1R signaling [18,19,23]. However, the mechanisms by which neutrophils contribute to the resolution of *L*. *pneumophila* lung infection remain incompletely understood.

IL-1 is closely linked to the induction of TNF in a broad spectrum of unrelated models of inflammation, and these cytokines are known to have synergistic effects *in vivo* [24–26]. Indeed, anti-TNF therapy is a recognized risk factor for Legionnaire's disease, suggesting a role for TNF in the immune response to *L*. *pneumophila* [27–31]. Previous work has established that TNF is produced in response to *L*. *pneumophila* in a T4SS-dependent and flagellin-independent manner [32,33] and can limit replication in macrophages [34–36]. Furthermore, it was shown that TNF contributes to immune defense against *L*. *pneumophila in vivo* [37–39]. However, the mechanisms by which TNF contributes to innate immune control of *L*. *pneumophila* and the cells upon which it acts *in vivo* have yet to be elucidated.

Macrophages from C57BL/6 mice are not permissive for *L*. *pneumophila* replication due to the intracellular sensor NAIP5 which binds cytosolic flagellin and recruits NLRC4, resulting in inflammasome assembly and the activation of Caspase-1 [40,41]. Active caspase-1 can initiate a pro-inflammatory form of cell death known as pyroptosis, the secretion of IL-1β and IL-18, as well as activate Caspase-7, which induces the fusion of lysosomes with LCVs, resulting in bacterial degradation [42,43]. Murine macrophages missing key components in this pathway are permissive to *L*. *pneumophila* replication, including NAIP5^-/-^, NLRC4^-/-^, Caspase-1^-/-^ and Caspase-7^-/-^ macrophages [42]. NLRC4 also restricts *L*. *pneumophila* via caspase-1 independent mechanisms [44]. Similarly, it has been shown that human NAIP (hNAIP), the only NAIP protein identified in humans, can mediate inflammasome assembly and *L*. *pneumophila* restriction when overexpressed in murine macrophages, and that *L*. *pneumophila* replication is enhanced in human macrophages when hNAIP is silenced [45,46]. Furthermore, primary human macrophages sense *L*. *pneumophila* flagellin via hNAIP and activate caspase-1 [47,48].

Macrophages from A/J mice are permissive to *L*. *pneumophila* replication due to an allelic variation in the NAIP5 gene, resulting in 14 amino acid (aa) differences as compared to C57BL/6 mice [49,50]. A/J macrophages are able to activate Caspase-1 in response to *L*. *pneumophila* infection [51], but fail to activate caspase-7, suggesting that at least some of the 14 aa are involved in promoting caspase-1 and caspase-7 interactions [40,42]. Other mouse strains also display partial susceptibility to *L*. *pneumophila* infection and replication, including FvB/N, C3H/HeJ, BALB/cJ and 129S1 mice [49]. In this paper we make use of mice with the 129S1 NAIP5 allele (NAIP5^129S1^) that have a targeted TNF deletion in macrophages, monocytes and neutrophils (MN-TNF NAIP5^129S1^ mice) [52] to examine the role of TNF derived from macrophages, monocytes and neutrophils in *L*. *pneumophila* lung infection in the absence of strong NAIP5 signaling.

In the present study, we demonstrate that TNF and reactive oxygen species (ROS) are essential for the effective innate immune control of *L*. *pneumophila*, and that *in vivo* TNF can compensate for the well characterized NLRC4-NAIP5 flagellin pathway. While ROS are essential for the bactericidal activity of neutrophils, TNF produced by neutrophils and monocytes is required to enhance AM-mediated restriction of *L*. *pneumophila* via TNFR1 *in vivo*. This TNF-mediated antibacterial mechanism is independent of NLRC4, caspase-1 and 11, but involves other caspases with a minor contribution from cysteine-type cathepsins or calpains, and also the fusion of LCVs with lysosomes and their acidification. The striking susceptibility of MN-TNF NAIP5^129S1^ mice to *L*. *pneumophila* lung infection suggests that TNF is a key component of innate immunity to *L*. *pneumophila* lung infection, especially when NAIP5-NLRC4 mediated responses are dampened.

## Results

### TNF and ROS are important for the clearance of *L*. *pneumophila in vivo*

**Fig 1:**
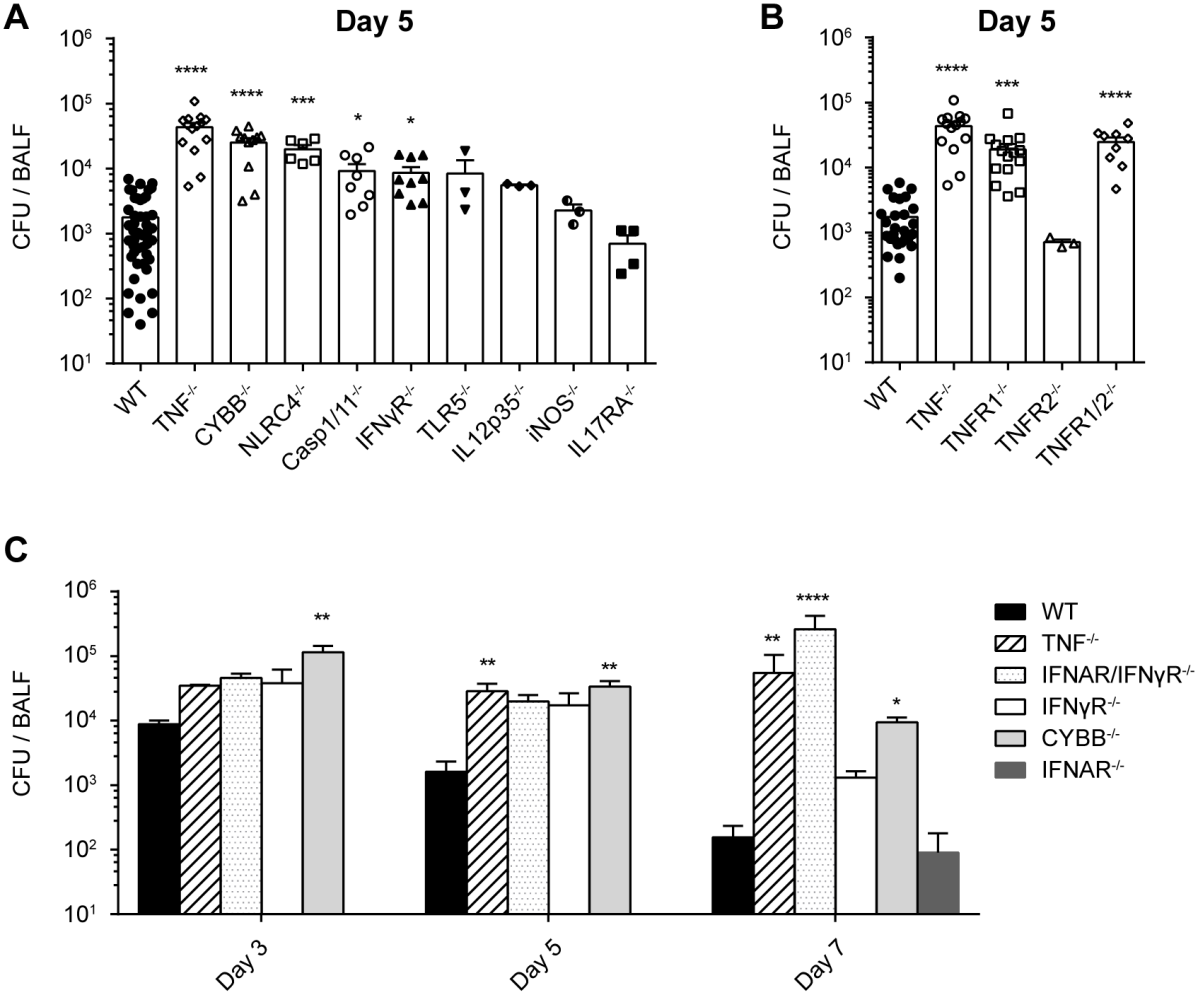
TNF / TNFR1 and ROS are important for clearance of *L*. *pneumophila in vivo*. **(A-C)** WT or knockout mice were infected intranasally with WT *L*. *pneumophila*, and 5 days p.i. (A) or 3–7 days p.i. (C) BALF CFU were quantified on CYE agar plates. Data in panel A, B and C are from 15, 8, and 2 pooled experiments, respectively. *p<0.05, **p<0.01, ***p<0.001, ****p<0.0001 compared to WT by Kruskal-Wallis test with Dunn's post test.

Many host immune factors have been shown to be involved in *L*. *pneumophila* control *in vitro*, whereas relatively few studies have assessed their impact *in vivo*. We therefore used an intranasal mouse infection model to identify crucial innate immune effector molecules and pathways that have been implicated in the clearance of *L*. *pneumophila* lung infection, by assessing their relative impact on bacterial burden in the lung 3–7 days p.i.. As has been previously demonstrated, we found that while IFNγR^-/-^ and IFNAR^-/-^ mice showed limited susceptibility to infection, double deficiency for IFNAR/IFNγR dramatically increased bacterial loads, in particular by day 7 post infection (Fig 1A and 1C, [53]). Similarly, by day 5 and 7 p.i., TNF deficiency resulted in severely increased bacterial burden, and deficiency in the phagocyte NADPH oxidase NOX2/gp91^phox^ (CYBB^-/-^ mice) resulted in potent impairment in bacterial control from day 3 through to day 7 (Fig 1A and 1C). In contrast, NLRC4, caspase-1/11, TLR5, IL-12, iNOS and IL17RA seem to play a less dominant role in controlling *L*. *pneumophila* lung infection (Fig 1A). These results show that TNF and ROS, as well as the combined action of Type I and II IFN signaling are crucial for the innate immune response to *L*. *pneumophila* lung infection.

### TNF acts via TNFR1 to control *L*. *pneumophila* infection

To identify the receptor through which TNF exerts its protective effect, WT, TNF^-/-^, TNFR1^-/-^, TNFR2^-/-^ and TNFR1/2^-/-^ mice were infected intranasally with WT *L*. *pneumophila* and CFUs were compared in the BALF 5 days p.i.. Bacterial clearance was delayed to a similar extent in TNF^-/-^, TNFR1^-/-^ and TNFR1/2^-/-^ but not TNFR2^-/-^ mice compared to WT mice, showing that TNF mediates its antibacterial effect via TNFR1 *in vivo* (Fig 1B).

### TNF / TNFR1 signaling contributes to AM but not neutrophil-mediated killing of *L*. *pneumophila in vivo*

**Fig 2:**
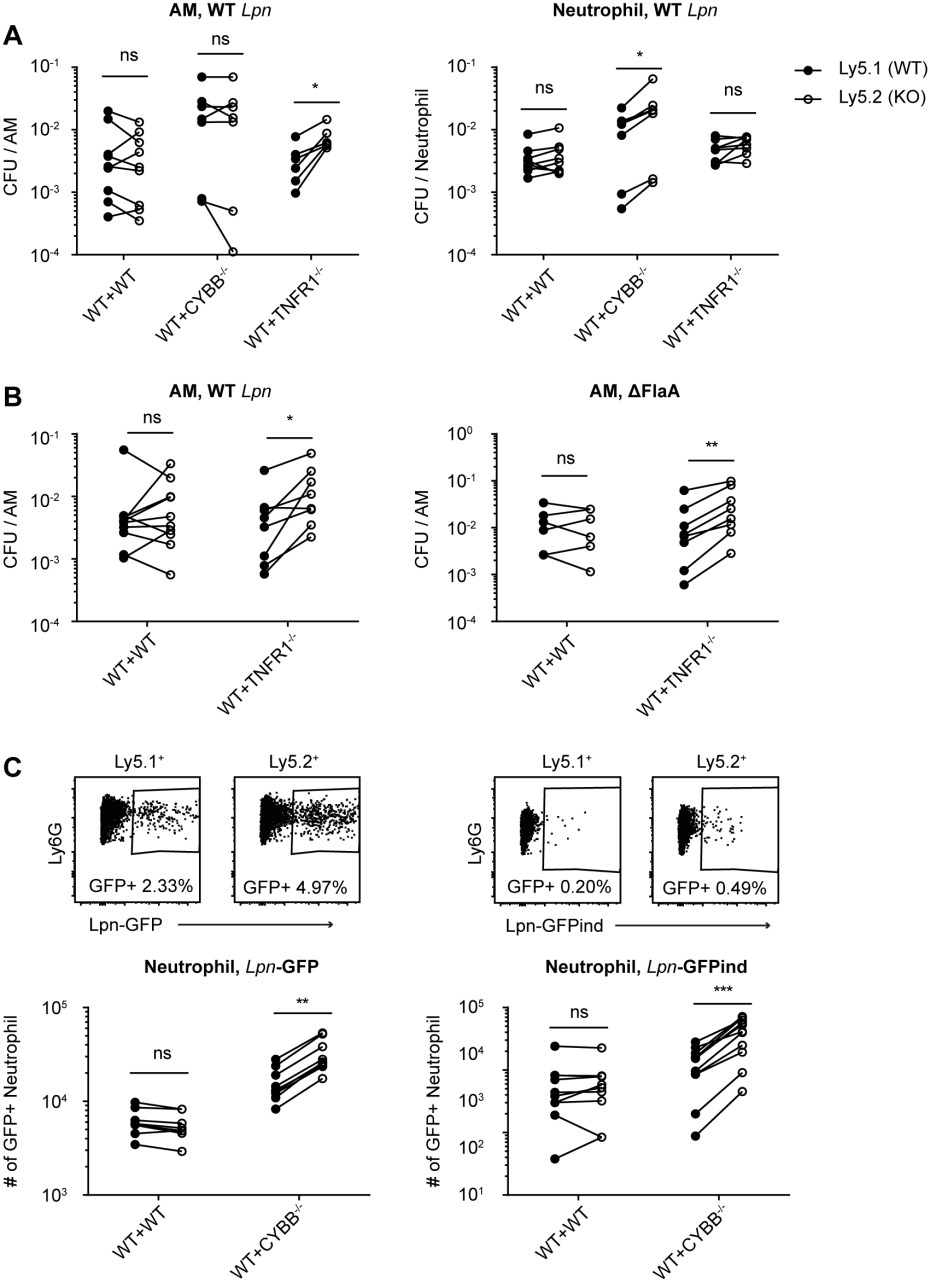
TNF / TNFR1 signaling contributes to AM-mediated killing of *L*. *pneumophila*, while ROS are required for efficient neutrophil-mediated killing *in vivo*. **(A-C)** Mixed BM chimeric mice reconstituted with 50% Ly5.1^+^ WT BM, and either 50% Ly5.2^+^ WT, TNFR1^-/-^ or CYBB^-/-^ BM were generated. **(A)** Chimeras were infected with WT *L*. *pneumophila*, and 2 days p.i. BALF was harvested and Ly5.1^+^ and Ly5.2^+^ AM and neutrophils were sorted. Cells were lysed and CFU were quantified on CYE agar plates. **(B)** Chimeras were infected with WT or ΔFlaA *L*. *pneumophila*, and CFU were quantified in AM as in A). **(C)** Chimeras were infected with *Lpn*-GFP or *Lpn*-GFPind (with IPTG induction) and BALF was analyzed by flow cytometry 38 hr p.i.. GFP^+^ neutrophils were normalized for the number of Ly5.1^+^ and Ly5.2^+^ neutrophils, respectively. Data are from 2–4 pooled experiments. *p<0.05, **p<0.01, ***p<0.001 by Wilcoxon test.

A recent study using a T4SS-based reporter system has demonstrated that AM and neutrophils are the primary targets for *L*. *pneumophila in vivo*, with *L*. *pneumophila* replication having been demonstrated in AM [10]. We therefore examined the impact of TNF on AM and neutrophil-mediated killing of *L*. *pneumophila in vivo*. To circumvent the problem that TNFR1^-/-^ mice have greater bacterial burdens in the lung than WT mice and allow for the direct comparison of AM and neutrophil bacterial loads in WT and TNFR1^-/-^ cells within a single mouse, we used a mixed chimera approach. Mixed bone marrow (BM) chimeric mice were generated with a mix of 50% Ly5.1^+^ WT BM and either 50% Ly5.2^+^ WT or Ly5.2^+^ TNFR1^-/-^ BM. After 8 weeks of reconstitution, WT:WT and WT:TNFR1^-/-^ mice were inoculated intranasally with WT *L*. *pneumophila*, and 2 days p.i. Ly5.1^+^ and Ly5.2^+^ AM and neutrophils were sorted from the BALF, and cells were plated on CYE plates to quantify viable *L*. *pneumophila*. Significantly more CFU / AM were recovered from TNFR1^-/-^ AM than from WT AM, indicating that TNF signaling via TNFR1 promotes the killing of *L*. *pneumophila* by AM *in vivo* (Fig 2A and 2B). In contrast, there was no difference in the number of viable *L*. *pneumophila* / neutrophil recovered from WT vs. TNFR1^-/-^ neutrophils, indicating that TNF signaling does not contribute to neutrophil-mediated killing of *L*. *pneumophila* (Fig 2A). The killing of *L*. *pneumophila* lacking flagellin was also impaired in TNFR1^-/-^ AM compared to WT AM, demonstrating that the antibacterial mechanism mediated in AM by TNF / TNFR1 is independent of the NAIP5-NLRC4 flagellin recognition pathway (Fig 2B). These results highlight that TNF / TNFR1 signaling mediates a non-redundant antibacterial mechanism that contributes to *L*. *pneumophila* killing in AM but not in neutrophils *in vivo*.

### ROS are required for efficient neutrophil but not AM-mediated killing of *L*. *pneumophila in vivo*

To analyze the impact of ROS on AM and neutrophil-mediated killing of *L*. *pneumophila*, we generated BM chimeric mice with a mix of 50% Ly5.1^+^ WT BM and either 50% WT Ly5.2^+^ or Ly5.2^+^ CYBB^-/-^ BM. 2 days p.i. we observed that while sorted CYBB^-/-^ AM did not contain more viable *L*. *pneumophila* / AM than WT AM, sorted CYBB^-/-^ neutrophils contained more viable *L*. *pneumophila* / neutrophil than did WT neutrophils from the same mouse (Fig 2A). This indicates that in contrast to TNF, NOX2-derived ROS play a non-redundant role in neutrophil-mediated killing of *L*. *pneumophila* but not AM-mediated killing of *L*. *pneumophila in vivo*.

We performed similar experiments in which WT:WT and WT:CYBB^-/-^ BM chimeric mice were inoculated with either *L*. *pneumophila* constitutively expressing GFP (*Lpn*-GFP), or with *L*. *pneumophila* containing a plasmid on which GFP expression can be induced by the addition of IPTG (*Lpn*-GFPind), thereby identifying metabolically active bacteria (Fig 2C). Neutrophils were analyzed by flow cytometry 38 hours p.i., and in the case of *Lpn* -GFPind infected mice, IPTG was administered intranasally at 35 hours p.i., resulting in the induction of GFP in all viable *L*. *pneumophila*. In line with the results of the BM chimera sort and plating experiments, there were more GFP^+^ CYBB^-/-^ neutrophils than GFP^+^ WT neutrophils in WT:CYBB^-/-^ BM chimeric mice, both with *Lpn*-GFP infection and with *Lpn*-GFPind infection (Fig 2C). In the case of *Lpn*-GFP infection this indicates that there were more NOX2-deficient neutrophils that contained dead or viable *L*. *pneumophila* than WT neutrophils, and in the case of *Lpn*-GFPind infection it indicates that there were more NOX2-deficient neutrophils that contained viable *L*. *pneumophila* than WT neutrophils in the same mouse. These data support the hypothesis that neutrophils require ROS to kill and degrade *L*. *pneumophila in vivo*.

### *In vivo*, neutrophils but not AM produce ROS in response to *L*. *pneumophila* infection

**Fig 3:**
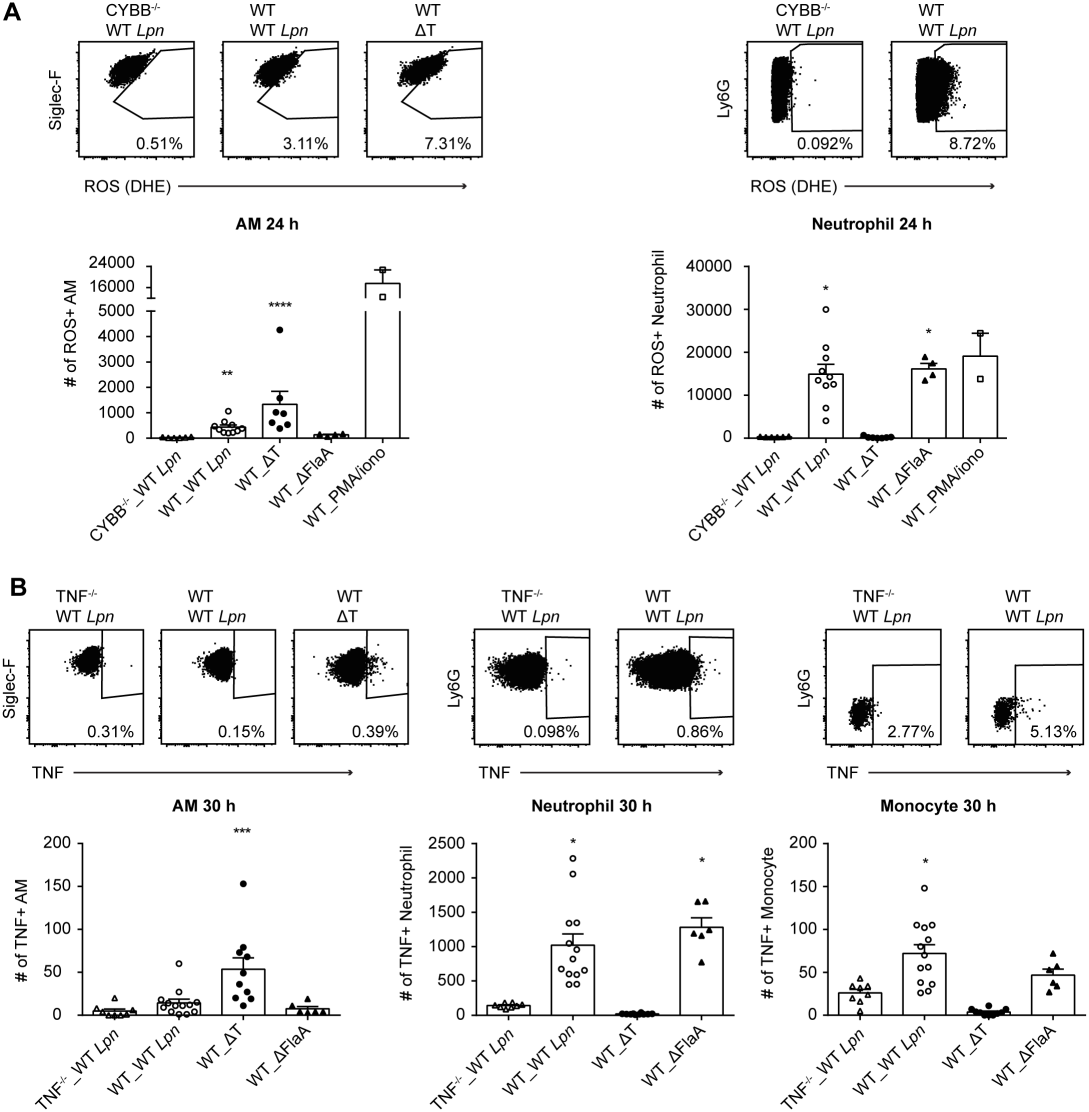
*In vivo*, neutrophils but not AM produce ROS and TNF in response to *L*. *pneumophila* infection. **(A)** WT and CYBB^-/-^ mice were infected intranasally with WT, ΔT or ΔFlaA *L*. *pneumophila* and BALF cells were harvested 24 hr p.i.. AM, neutrophils and monocytes were stained for ROS with Dihydroethidium (DHE) and analyzed by flow cytometry. **(B)** WT and TNF^-/-^ mice were infected intranasally with WT, ΔT or ΔFlaA *L*. *pneumophila* and BALF cells were harvested 30 hr p.i.. AM, neutrophils and monocytes were stained for TNF and analyzed by flow cytometry. Data are from 2–3 pooled experiments. *p<0.05, **p<0.01, ***p<0.001, ****p<0.0001 compared to CYBB^-/-^ or TNF^-/-^ mice by Kruskal-Wallis test with Dunn's post test.

Having established that NOX2-dependent mechanisms are involved in neutrophil-mediated killing of *L*. *pneumophila*, we sought to determine if neutrophils actively produce ROS in response to *L*. *pneumophila*. We infected WT and CYBB^-/-^ mice with WT, T4SS deficient (ΔT) and ΔFlaA *L*. *pneumophila* and stained neutrophils and AM with a flow cytometry based ROS detection reagent (Dihydroethidium) 24 h p.i.. We observed that neutrophils but not AM produced ROS in response to WT and ΔFlaA *L*. *pneumophila* 24 h p.i., suggesting that ROS could have direct bactericidal effects in *L*. *pneumophila* containing neutrophils (Fig 3A). Since we did not observe neutrophil ROS production in response to ΔT *L*. *pneumophila*, our results suggest this ROS production is T4SS-dependent and flagellin independent (Fig 3A). Conversely, AM produced very little ROS in response to WT *L*. *pneumophila*, but more in response to ΔT *L*. *pneumophila*, in line with a publication suggesting that *L*. *pneumophila* actively inhibits ROS production in macrophages via T4SS-dependent effector molecules (Fig 3A, [54]).

### TNF mediates an antibacterial effect in macrophages via TNFR1, which is independent of NLRC4 and NOX2

**Fig 4:**
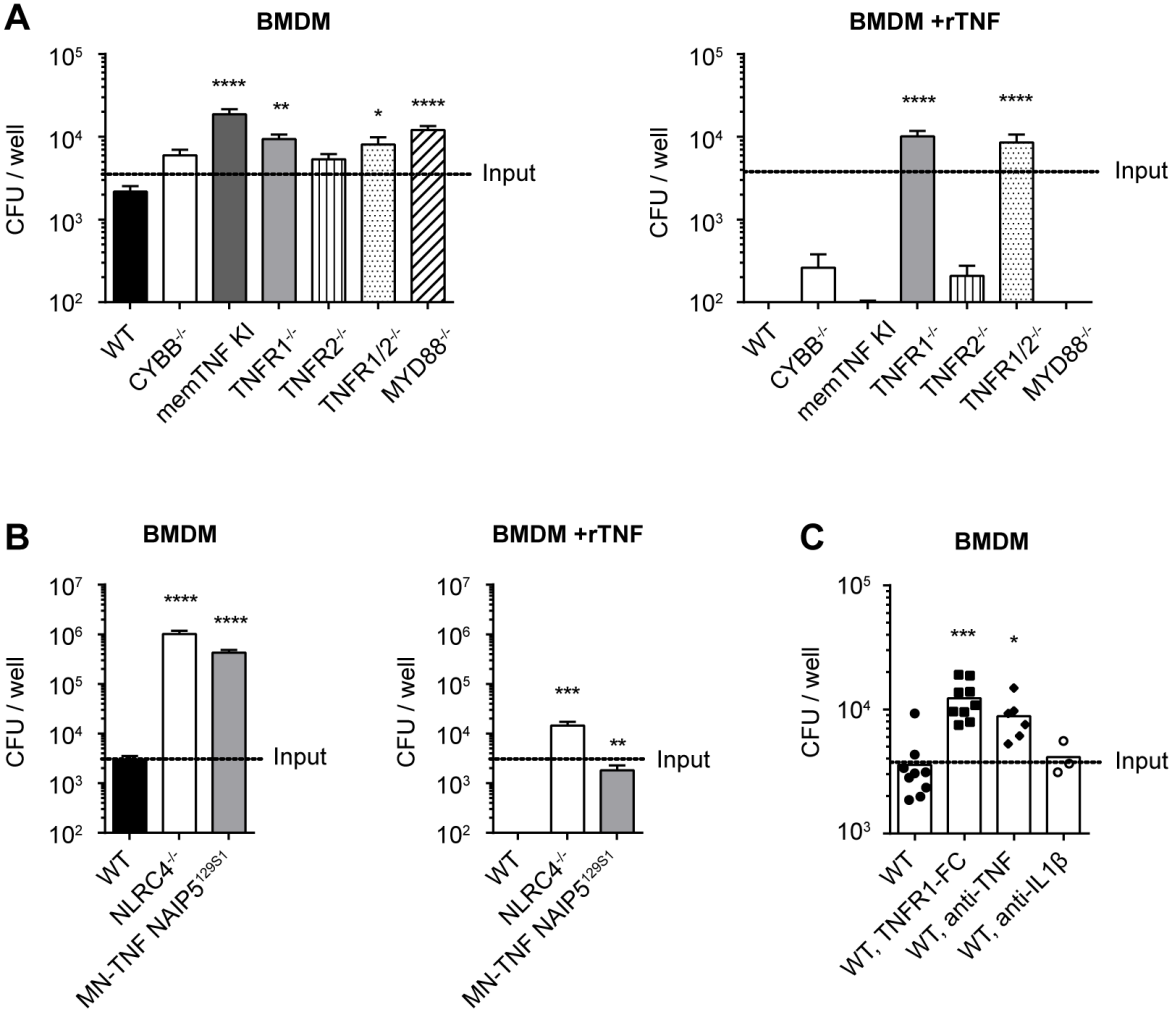
TNF mediates an antibacterial effect in macrophages via TNFR1, which is independent of NLRC4 and ROS. **(A-B)** WT or knockout BMDM were infected with WT *L*. *pneumophila* at MOI 0.1. BMDM were either left untreated (left hand panels) or rTNF was added at the time of infection (right hand panels). 3 days p.i. BMDM were lysed and CFU were quantified on CYE agar plates. **(C)** WT BMDM were infected with WT *L*. *pneumophila* MOI 0.1, with or without the addition of TNFR1-Fc, anti-TNF Ab or anti-IL1β Ab. 3 days p.i. BMDM were lysed and CFU were quantified on CYE agar plates. Data are from 3–7 pooled experiments. *p<0.05, **p<0.01, ***p<0.001, ****p<0.0001 compared to WT by Kruskal-Wallis test with Dunn's post test.

The *in vivo* results presented in Fig 2A in combination with the observation that *in vitro*, TNFR1^-/-^ and TNFR1/2^-/-^ but not TNFR2^-/-^ bone marrow derived macrophages (BMDM) were more permissive to *L*. *pneumophila* replication than WT BMDM, suggest that TNF directly inhibits *L*. *pneumophila* replication in macrophages via signaling through TNFR1 (Fig 4A). Furthermore, the addition of recombinant TNF (rTNF) to BMDM abrogated *L*. *pneumophila* growth in all of the genotypes with a functional TNFR1, including NLRC4^-/-^ and CYBB^-/-^ BMDM (Fig 4A). These data show that TNF-mediates an antibacterial mechanism in BMDM via TNFR1, which is independent of NOX2-derived ROS and the NAIP5-NLRC4 flagellin recognition pathway. The flagellin independence of this mechanism was further shown by the TNF-mediated abrogation of ΔFlaA *L*. *pneumophila* replication in WT BMDM but not TNFR1^-/-^ BMDM (S3B Fig). Importantly, three day exposure to 100 ng/ml rTNF did not induce BMDM cell death (S1 Fig), suggesting an active antibacterial mechanism mediated by TNF rather than the induction of cell death. Membrane TNF knock-in (memTNF KI) BMDM, which are only able to make membrane bound but not secreted TNF, were also more susceptible than WT BMDM, suggesting that TNF signals as a soluble molecule on BMDM *in vitro* (Fig 4A). To consolidate this observation, we added a neutralizing anti-TNF antibody or TNFR1 fused to the Fc portion of human IgG1 (TNFR1-Fc) to WT BMDM infected with *L*. *pneumophila*, in order to neutralize soluble TNF secreted by the BMDM. This resulted in the sensitization of WT BMDM to *L*. *pneumophila* infection to a similar level as that observed for TNFR1^-/-^ BMDM, suggesting that the difference in susceptibility between WT and TNFR1^-/-^ BMDM is due to endogenously secreted TNF in response to *L*. *pneumophila* infection (Fig 4A and 4C). Also in line with the conclusion that lack of endogenous TNF results in moderate sensitivity of BMDM to *L*. *pneumophila* infection is the observation that MyD88^-/-^ BMDM, which fail to secrete TNF in response to *L*. *pneumophila* infection (S2 Fig and [36]), also have a similar susceptibility to *L*. *pneumophila* as TNFR1^-/-^ BMDM (Fig 4A). Taken together, these data suggest that TNF activates an antibacterial mechanism in macrophages via TNFR1 that is independent of NLRC4 and NOX2. Furthermore, TNF production by BMDM in response to *L*. *pneumophila* is downstream of MyD88.

### *In vivo*, TNF produced by neutrophils and monocytes enhances AM-mediated killing of *L*. *pneumophila*

In order to determine which cells produce TNF *in vivo*, we infected WT mice and TNF^-/-^ mice with WT, ΔT and ΔFlaA *L*. *pneumophila* and stained BALF cells for TNF 30 hr p.i.. We found that neutrophils and monocytes produced TNF in response to *L*. *pneumophila* lung infection, suggesting that neutrophils and monocytes are the relevant TNF source (Fig 3B).

**Fig 5:**
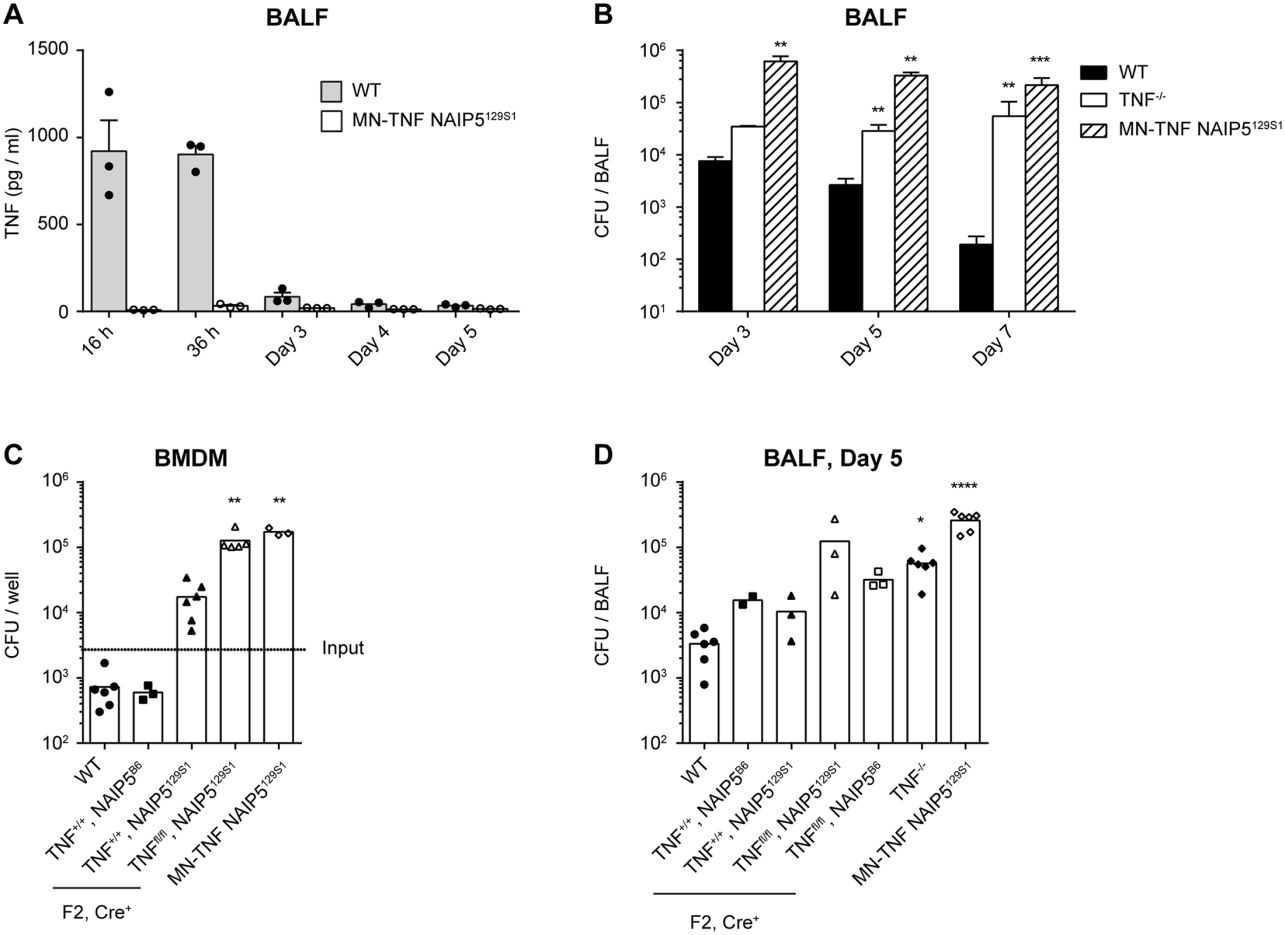
NAIP5^129S1^ and TNF-deficiency in macrophages, monocytes and neutrophils are the genetic traits that render MN-TNF NAIP5^129S1^ mice susceptible to *L*. *pneumophila* infection *in vitro* and *in vivo*. **(A-B)** WT, TNF^-/-^, MN-TNF NAIP5^129S1^ mice were infected intranasally with WT *L*. *pneumophila*, and analyzed at the indicated time points. **(A)** TNF was quantified in the BALF via CBA assay. **(B)** BALF CFU were quantified on CYE agar plates. **(C)** WT or MN-TNF NAIP5^129S1^ BMDM, or BMDM from the F2 offspring of MN-TNF NAIP5^129S1^ x C57BL/6 intercrosses were infected with WT *L*. *pneumophila* at MOI 0.1. 3 days p.i. BMDM were lysed and CFU were quantified on CYE agar plates. **(D)** WT, TNF^-/-^, MN-TNF NAIP5^129S1^ mice, or the F2 offspring of MN-TNF NAIP5^129S1^ x C57BL/6 intercrosses were infected intranasally with WT *L*. *pneumophila*, and 5 days p.i. BALF CFU were quantified on CYE agar plates. All of the F2 offspring shown are Cre^+^. Panel A is from 1 experiment, panels B-D are from 2–3 pooled experiments. *p<0.05, **p<0.01, ***p<0.001, ****p<0.0001 compared to WT by Kruskal-Wallis test with Dunn's post test.

As has been shown in published results, we observed that NLRC4^-/-^ mice were only moderately susceptible to infection, despite the well-recognized role of NLRC4 in inflammasome activation in response to *L*. *pneumophila* flagellin, and the high susceptibility of NLRC4^-/-^ macrophages to *L*. *pneumophila* replication *in vitro* (Figs 1A and 4B, [55], [44]). The fact that NLRC4^-/-^ BMDM are highly susceptible to *L*. *pneumophila* replication *in vitro*, but NLRC4^-/-^ mice are only moderately susceptible *in vivo*, suggests that mechanisms that are only present *in vivo* are able to compensate for a lack of NLRC4. To determine if paracrine TNF compensates for a lack of NAIP5-NLRC4-mediated signaling *in vivo*, we infected MN-TNF NAIP5^129S1^ mice, which have a hypofunctional NAIP5 allele (NAIP5^129S1^) and are deficient in TNF in macrophages, monocytes and neutrophils, with WT *L*. *pneumophila*. We found that MN-TNF NAIP5^129S1^ mice were highly susceptible to *L*. *pneumophila* lung infection, with much greater bacterial burdens in the BALF 5 days p.i. compared to WT mice, and also compared to TNF^-/-^ and NLRC4^-/-^ mice (Figs 1A and 5B). Taken together, these results suggests that TNF produced by neutrophils and monocytes is essential for *in vivo* control of *L*. *pneumophila* lung infection. In addition, BMDM from MN-TNF NAIP5^129S1^ mice were almost as susceptible to *L*. *pneumophila* replication as NLRC4^-/-^ BMDM, and this susceptibility could be abrogated by the addition of rTNF (Fig 4B). Taken together, these data suggest that neutrophil and monocyte derived TNF enhances AM-mediated *L*. *pneumophila* killing and partially compensates for a lack of NAIP5-NLRC4 signaling in AM *in vivo*.

To verify that TNF is indeed abrogated in MN-TNF NAIP5^129S1^ mice, we infected WT and MN-TNF NAIP5^129S1^ mice intranasally with WT *L*. *pneumophila*, and measured TNF in the BALF (Fig 5A). MN-TNF NAIP5^129S1^ mice had almost undetectable TNF in the BALF following intranasal *L*. *pneumophila* infection, confirming that in the context of intranasal *L*. *pneumophila* lung infection, neutrophils and monocytes are the primary source of TNF.

Since 129 mice have a documented mutation in caspase-11 [56], we sequenced this gene in MN-TNF NAIP5^129S1^ mice and found it to be WT. To rule out the possibility that further genes besides NAIP5 from the 129S1 genetic background influenced the phenotype of MN-TNF NAIP5^129S1^ mice in our experiments, we backcrossed them to C57BL/6 mice, and used the F2 offspring to conduct littermate controlled experiments. All the offspring we used for experiments were positive for MLys-Cre and the NAIP5 locus was sequenced for each individual mouse. We compared *in vitro L*. *pneumophila* replication in BMDM derived from the homozygous F2 offspring (TNF^+/+^/NAIP5^B6^, TNF^+/+^/NAIP5^129S1^, TNF^fl/fl^/NAIP5^B6^, TNF^fl/fl^/NAIP5^129S1^), C57BL/6 (WT), TNF^-/-^ and MN-TNF NAIP5^129S1^ mice as well as *in vivo* bacterial loads in the BALF 5 days after intranasal *L*. *pneumophila* infection (Fig 5C and 5D). We observed that BMDM from F2 offspring that were TNF sufficient and carried the NAIP5^B6^ allele were as resistant to WT *L*. *pneumophila* infection as WT BMDM, indicating that these two genes were responsible for the enhanced susceptibility of MN-TNF NAIP5^129S1^ BMDM (Fig 5C). Furthermore, TNF^+/+^/NAIP5^129S1^ BMDM supported more *L*. *pneumophila* growth than did TNF^+/+^/NAIP5^B6^ BMDM, though this was not statistically significant, and TNF^fl/fl^/NAIP5^129S1^ BMDM were as susceptible as MN-TNF NAIP5^129S1^ BMDM (Fig 5C). These data suggest that MN-TNF NAIP5^129S1^ BMDM are more susceptible to *L*. *pneumophila* infection than WT BMDM due to defects in both NAIP5 signaling and TNF production.

*In vivo*, we found that TNF^+/+^ littermates with NAIP5^129S1^ were not more susceptible than TNF^+/+^ littermates with NAIP5^B6^, which is in line with our previous findings and published data indicating that reduced NAIP5-NLRC4 signaling has only a moderate impact on susceptibility to *L*. *pneumophila* lung infection *in vivo* (Fig 5D). In contrast, TNF^fl/fl^/NAIP5^129S1^ mice tended to have greater susceptibility to *L*. *pneumophila* lung infection, similar to MN-TNF NAIP5^129S1^ mice, while TNF^fl/fl^/NAIP5^B6^ mice had similar susceptibility to TNF^-/-^ mice (Fig 5D). These data suggest that NAIP5^129S1^ and TNF deficiency in macrophages, monocytes and neutrophils are the genetic elements that mediate the enhanced susceptibility of MN-TNF NAIP5^129S1^ mice to *L*. *pneumophila* lung infection.

### TNF induces the fusion of LCVs with lysosomal compartments in macrophages

**Fig 6:**
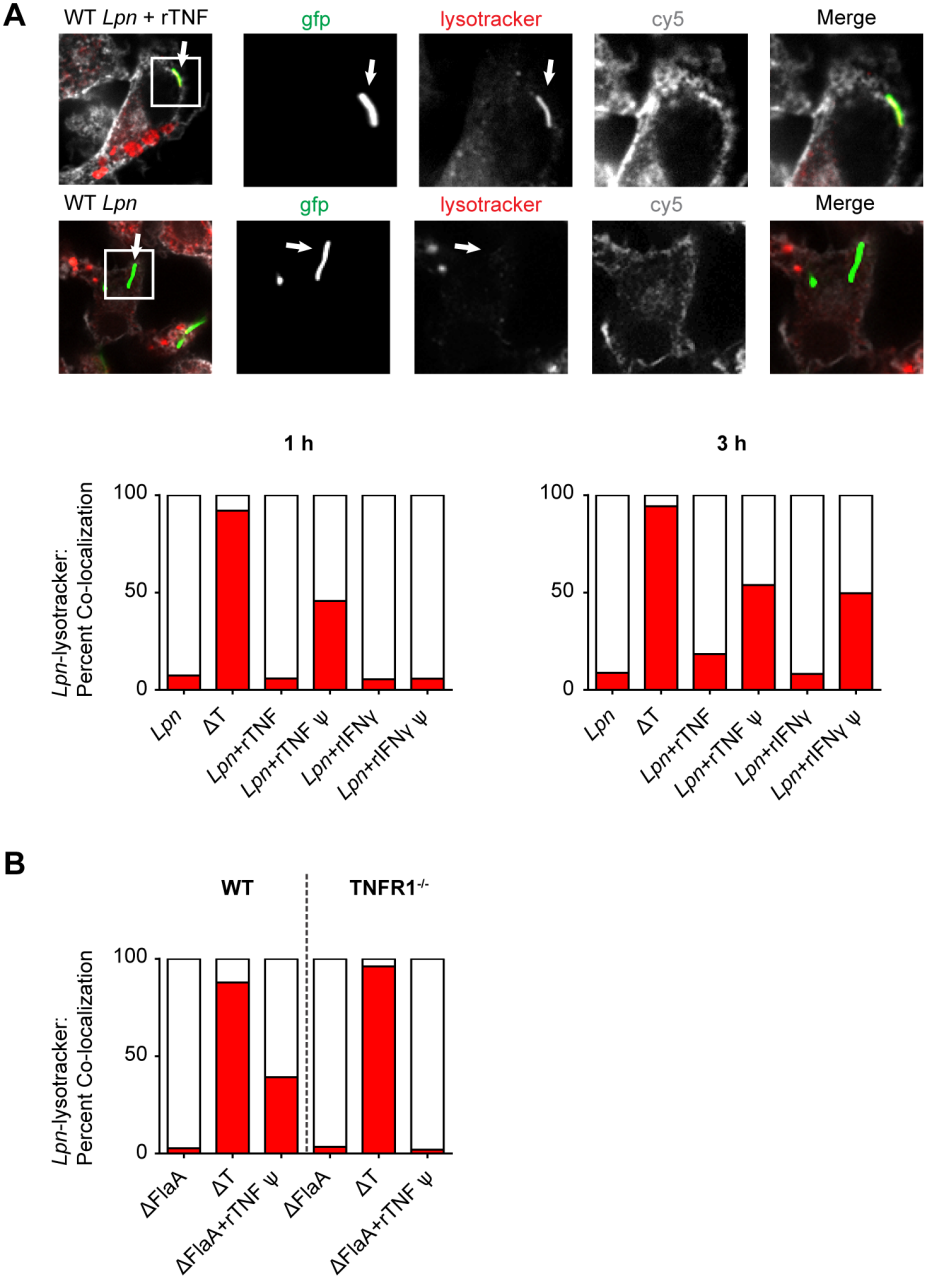
TNF-mediated killing of *L*. *pneumophila* is associated with the fusion of LCVs with lysosomal compartments in macrophages. **(A)** MN-TNF NAIP5^129S1^ BMDM were pre-treated overnight with rTNF (rTNFѱ), rIFNγ (rIFNγѱ), or were left untreated, and then infected with *Lpn*-GFP or ΔT-GFP at MOI 5 with simultaneous addition of rTNF or rIFNγ where indicated. 1 hr or 3 hr p.i. co-localization of *Lpn*-GFP with lysosomes (stained with lysotracker Red) was analyzed via confocal microscopy, and at least 100 bacteria were counted per group. BMDM cell membranes were stained with Cholera toxin B AF647 (cy5). Data are representative of 2 experiments. **(B)** WT or TNFR1^-/-^ BMDM were pre-treated overnight with rTNF (rTNFѱ) or were left untreated, and then infected with ΔFlaA *Lpn*-GFP or ΔT-GFP at MOI 5 with simultaneous addition of rTNF where indicated. 1 hr p.i. co-localization of *Lpn*-GFP with lysosomes (stained with lysotracker Red) was analyzed via confocal microscopy as in A) and at least 100 bacteria were counted per group. Data is from 1 experiment.

To gain further insight into the antibacterial mechanism mediated by TNF in macrophages, we infected MN-TNF NAIP5^129S1^ BMDM with *Lpn*-GFP, in the presence or absence of rTNF or rIFNγ as a positive control [57], and examined the fate of the LCV with respect to lysosomal fusion using confocal microscopy. By 3 hours p.i. neither 100 ng/ml rTNF nor 200 U/ml rIFNγ resulted in *Lpn*-GFP co-localization with lysosomal compartments as defined by lysotracker staining (Fig 6A). However, when MN-TNF NAIP5^129S1^ BMDM were pre-treated with rTNF or rIFNγ overnight, by 1 hour p.i. 50% of *L*. *pneumophila* in rTNF pre-treated MN-TNF NAIP5^129S1^ BMDM co-localized with lysosomal compartments, but not in rIFNγ pre-treated BMDM. By 3 hours p.i., *Lpn*-GFP co-localization with lysosomal compartments was observed in both rTNF and rIFNγ pre-treated MN-TNF NAIP5^129S1^ BMDM, suggesting that TNF induces the fusion of lysosomes with the LCV, but with different kinetics than IFNγ (Fig 6A). Furthermore, pre-treatment with rTNF was also shown to induce co-localization of ΔFlaA *Lpn* in WT BMDM but not TNFR1^-/-^ BMDM after 1 hr, confirming TNF-mediated flagellin-independent induction of the fusion of lysosomes with LCVs in macrophages (Fig 6B).

### The antibacterial mechanism mediated by TNF in macrophages is dependent on lysosomal acidification and caspase activity

**Fig 7:**
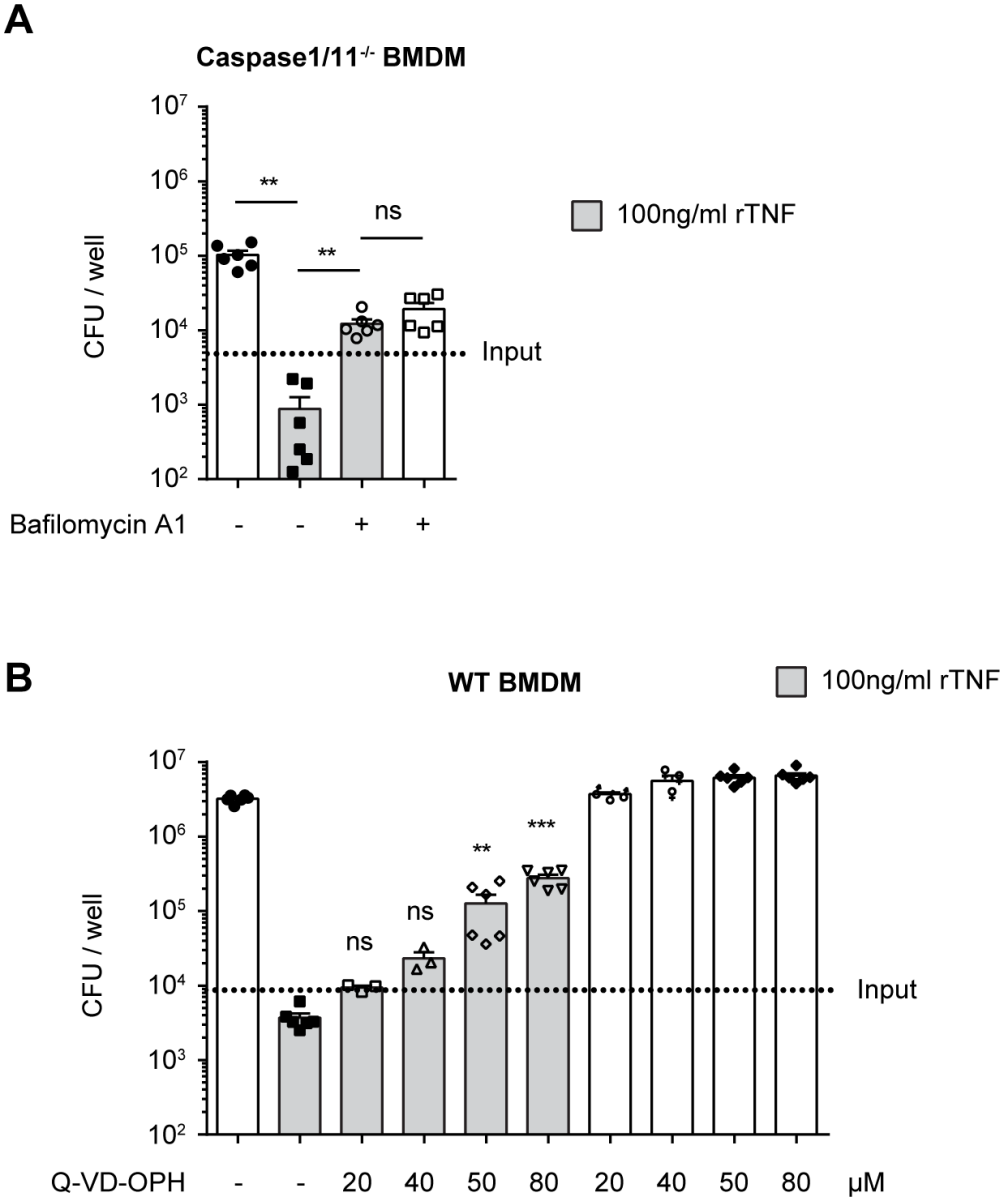
TNF-mediated killing of *L*. *pneumophila* is dependent of lysosomal acidification and caspases other than caspase-1 and 11 in macrophages. **(A)** Caspase-1/11^-/-^ BMDM were infected with WT *L*. *pneumophila* at MOI 0.1. Where indicated rTNF and/or v-ATPase inhibitor bafilomycin A1 were added at the time of infection. 3 days p.i. BMDM were lysed and CFU were quantified on CYE agar plates. Data are from 2 pooled experiments. **p<0.01 by Mann-Whitney test. **(B)** WT BMDM were either pre-treated with rTNF overnight or left untreated, and then infected with ΔFlaA *L*. *pneumophila* at MOI 0.1. Where indicated rTNF and/or pan caspase inhibitor Q-VD-OPh were added at the time of infection. 3 days p.i. BMDM were lysed and CFU were quantified on CYE agar plates. Data are from 2 pooled experiments. **p<0.01, ***p<0.001 compared to WT+rTNF by Kruskal-Wallis test with Dunn's post test.

Given that the fusion of LCVs with lysosomes has been shown to be induced by caspase-11, as well as caspase-1 in conjunction with caspase-7 [42,58], we wished to determine if the antibacterial effect mediated by TNF is dependent on Caspase-1 or 11. We therefore infected caspase-1/11^-/-^ BMDM with *L*. *pneumophila*, with or without the addition of rTNF, and CFU were quantified 3 days p.i.. The addition of rTNF prevented bacterial replication in Caspase-1/11^-/-^ BMDM, demonstrating that the TNF-mediated antibacterial mechanism in BMDM is independent of Caspase 1 and 11 (Fig 7A). Since our co-localization experiments suggested that TNF redirected *L*. *pneumophila* to lysosomal compartments, we sought to determine if lysosomal acidification was required for the TNF-mediated mechanism. To test this we infected Caspase-1/11^-/-^ BMDM with *L*. *pneumophila* with or without rTNF and in the presence or absence of bafilomycin A1, a vacuolar H^+^-ATPase (v-ATPase) inhibitor that blocks lysosomal acidification. We found that bafilomycin A1 abrogates the TNF-mediated inhibition of *L*. *pneumophila*, suggesting that lysosomal acidification is required downstream of TNF (Fig 7A).

To determine if further caspases were involved, we tested if the TNF-mediated inhibition of *L*. *pneumophila* growth could be blocked using the third generation pan caspase inhibitor Q-VD-OPh, which is highly potent and specific for caspases [59]. We infected WT BMDM with ΔFlaA *L*. *pneumophila*, in the presence of increasing concentrations of Q-VD-OPh, with or without rTNF, and quantified CFU 3 days p.i.. Q-VD-OPh blocked TNF-mediated growth restriction in a dose dependent manner, suggesting that caspases other than caspase-1 and 11 are required for the TNF-mediated restriction of *L*. *pneumophila* growth in BMDM (Fig 7B). In summary, our data show that the antibacterial mechanism mediated by TNF in BMDM is dependent on at least one caspase and lysosomal acidification, but is independent of Caspase-1 and 11.

### TNF-mediated restriction of *L*. *pneumophila* growth in macrophages is enhanced by cysteine-type cathepsins or calpains

**Fig 8:**
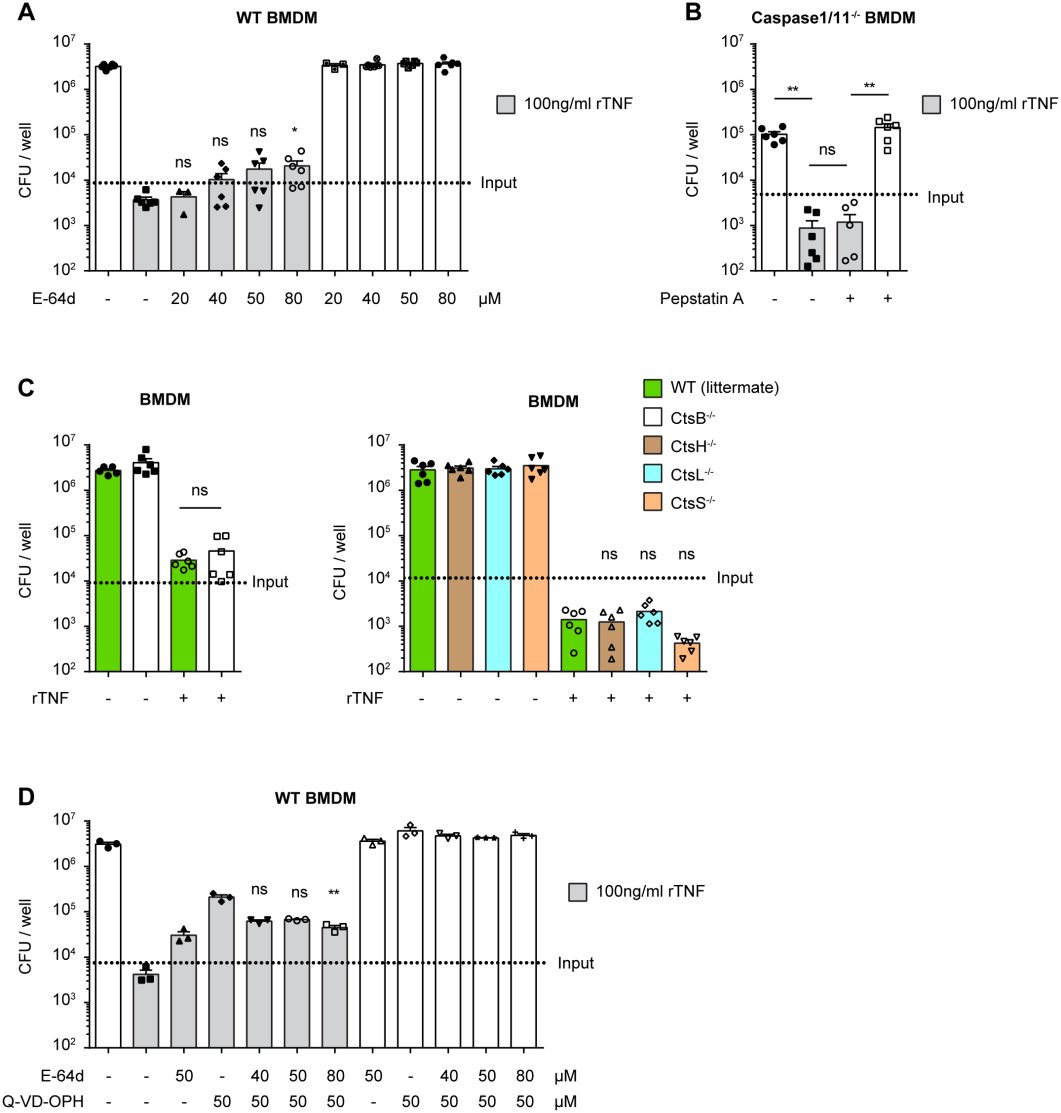
TNF-mediated restriction of *L*. *pneumophila* growth in macrophages is enhanced by cysteine-type cathepsins or calpains. **(A, C, D)** WT, CtsB^-/-^, CtsH^-/-^, CtsL^-/-^ or CtsS^-/-^ BMDM were either pre-treated with rTNF overnight or left untreated, and then infected with ΔFlaA *L*. *pneumophila* at MOI 0.1. Where indicated rTNF and/or E-64d and/or Q-VD-OPh were added at the time of infection. 3 days p.i. BMDM were lysed and CFU were quantified on CYE agar plates. **(B)** Caspase-1/11^-/-^ BMDM were infected with WT *L*. *pneumophila* at MOI 0.1. Where indicated rTNF and/or cathepsin D inhibitor pepstatin A were added at the time of infection. 3 days p.i. BMDM were lysed and CFU were quantified on CYE agar plates. (A) Data are from 2 pooled experiments. *p<0.05 compared to WT+rTNF by Kruskal-Wallis test with Dunn's post test. (B) Data are from 2 pooled experiments. **p<0.01 by Mann-Whitney test. (C) Data are from 2 pooled experiments. *p<0.05 by Mann-Whitney test (left panel) and *p<0.05 compared to WT by Kruskal-Wallis test with Dunn's post test. (D) Data are from 1 experiment. **p<0.01 compared to WT+rTNF+Q-VD-OPh by Kruskal-Wallis test with Dunn's post test.

To gain further insight into the TNF-induced bactericidal mechanisms responsible for *L*. *pneumophila* degradation in acidic compartments, we investigated the involvement of lysosomal proteases in the cathepsin family. For this we titrated the inhibitor E-64d, which potently inhibits cysteine-type cathepsins and calpains but not caspases [60–64], in the presence or absence of rTNF on ΔFlaA *L*. *pneumophila* infected WT BMDM. We observed that E-64d modestly reduced TNF-mediated restriction of *L*. *pneumophila* growth only at high concentrations, suggesting that cathepsins or calpains are involved in the TNF-mediated restriction of *L*. *pneumophila* growth in BMDM, but play a minor role (Fig 8A).

Next, we attempted to identify individual cathepsins involved in the TNF-mediated mechanism. Cathepsin D inhibition using pepstatin A did not interfere with the TNF-mediated restriction of *L*. *pneumophila* replication, suggesting that this aspartic protease is not required (Fig 8B). In follow up of the experiments with cysteine protease inhibitors described above, we observed that the pan caspase inhibitor Z-VAD-FMK partially blocked the TNF-mediated restriction of *L*. *pneumophila* growth, both in WT and caspase-1/11^-/-^ BMDM (S4 Fig). Since Z-VAD-FMK is also known to inhibit cathepsin B, H, L and S [59,60], we tested their involvement using BMDM from the corresponding cathepsin knockout mice. We found that rTNF suppressed *L*. *pneumophila* replication to a similar degree as in WT BMDM, showing that cathepsin B, H, L and S are not critical for TNF-mediated restriction of *L*. *pneumophila* growth in macrophages (Fig 8C). This, however, does not rule out that these cathepsins contribute redundantly to TNF-mediated inhibition of *L*. *pneumophila* growth in BMDMs.

To test if caspases and cathepsins / calpains have a synergistic role in TNF-mediated restriction of *L*. *pneumophila* replication, which could indicate that they are involved in separate converging pathways, we tested if the effect of E64d and Q-VD-OPh was additive. However, the concomitant addition of E-64d with Q-VD-OPh did not increase the ability of Q-VD-OPh to block the TNF-mediated effect (Fig 8D). These data suggest that caspases and cathepsins / calpains do not synergize to enhance TNF-mediated restriction of *L*. *pneumophila* growth, and could be involved interdependently in the same pathway. In conclusion, our data show that cathepsins or calpains contribute somewhat to TNF-mediated restriction of *L*. *pneumophila* replication in macrophages, but there is not a non-redundant requirement for cathepsin B, D, H, L or S. Therefore, among the proteases tested, the major cysteine proteases reducing the replication of *L*. *pneumophila* upon TNF treatment appear to be the caspases.

## Discussion

In this study, we identified cell-type specific key innate immune effector functions responsible for effective control of pulmonary *L*. *pneumophila* lung infection. Neutrophil-mediated mechanisms that lead to *L*. *pneumophila* clearance *in vivo* are twofold. On the one hand, neutrophils directly kill *L*. *pneumophila* via ROS-mediated mechanisms, and on the other hand, neutrophil and monocyte-derived TNF initiates microbicidal mechanisms in AM via TNFR1, which increase their capacity to inhibit *L*. *pneumophila* replication. The latter involves rerouting the bacteria to lysosomal compartments despite the presence of T4SS effectors, and requires at least one caspase other than caspase-1 or 11. The importance of TNF and NOX2-mediated mechanisms in the control of *L*. *pneumophila* infection are underscored by the marked susceptibility of TNF^-/-^ and CYBB^-/-^ mice to *L*. *pneumophila* infection.

The impact of TNF-mediated antimicrobial mechanisms directed against *L*. *pneumophila* cannot be fully appreciated by the study of macrophages *in vitro*. In accordance with other studies, we observed that TNFR1^-/-^ BMDM only support moderate *L*. *pneumophila* growth in comparison to NAIP5^-/-^ or NLRC4^-/-^ BMDM which support several orders of magnitude more growth (Fig 4A and 4B, [36,55]). However, this difference is not observed when comparing the bacterial burden of TNFR1^-/-^ and NLRC4^-/-^ mice *in vivo*, where there is even a trend for TNF to play a more dominant role (Fig 1A). A possible explanation for these apparently incongruent results is that paracrine TNF produced *in vivo* by neutrophils and monocytes, rather than autocrine TNF produced by AM, mediates the increased resistance to *L*. *pneumophila*, and further that this TNF can compensate for a lack of NAIP5-NLRC4-mediated immune defense. Though we and others did observe modest endogenous TNF production by BMDM in response to *L*. *pneumophila* infection (S2 Fig, [36]), and found that this TNF accounted for the increased susceptibility of TNFR1^-/-^ BMDMs (Fig 4C, [36]), this was not enough to compensate for lack of NAIP5-NLRC4 flagellin sensing (Figs 4B and S3B, [36]), arguing against a dominant role for autocrine TNF production by AM. In fact, NLRC4^-/-^ BMDM were highly susceptible to infection despite secreting more TNF than WT BMDM in response to *L*. *pneumophila* infection, possibly due to increased bacterial burden or a failure to undergo pyroptosis (S2 Fig, [36]). However, we propose that *in vivo*, AM are exposed to much higher local concentrations of TNF than produced endogenously by BMDM. *In vitro*, 200–600 pg/ml TNF were observed in the supernatant of *L*. *pneumophila* infected WT BMDM (S2 Fig, [36]), in comparison to 1 ng/ml we observed in the BALF (Fig 5A) and up to 20 ng/ml reported in the BALF at peak concentration [65,66]. Making the conservative estimate of an epithelial lining fluid volume of 100 μl, or a 10–20 fold dilution in 1–2 ml BALF, the actual TNF concentration in the undiluted endothelial lining fluid would be 10–400 ng/ml. Indeed, the addition of 100 ng/ml rTNF markedly suppressed *L*. *pneumophila* replication in NLRC4^-/-^ BMDM and increased cell viability (Figs 4B and S1). In addition, TNF has been shown to synergize with other cytokines such as IFNγ and type 1 interferons (IFN) in the restriction of *L*. *pneumophila*, which might also be present at higher concentrations in the epithelial lining fluid [34,36,53]. In line with this idea, bacterial burden is more severely impaired in TNF^-/-^ and IFNAR/IFNγR^-/-^ mice at later time points, which could reflect a shared mechanism of action (Fig 1B).

In order to verify the hypothesis that TNF might compensate for reduced NAIP5-NLRC4 mediated mechanisms, we made use of MN-TNF NAIP5^129S1^ mice, in which TNF is ablated in macrophages, monocytes and neutrophils and which carry the NAIP5^129S1^ allele. BMDM from MN-TNF NAIP5^129S1^ mice were almost as susceptible to *L*. *pneumophila* infection as BMDM from NLRC4^-/-^ mice, as expected in the absence of strong NAIP5 signaling (Fig 4B). Strikingly, MN-TNF NAIP5^129S1^ mice were also much more susceptible to *L*. *pneumophila* infection *in vivo* compared to either NLRC4^-/-^ or TNF^-/-^ mice, which in combination with the intracellular staining results suggests that neutrophil and monocyte derived TNF compensates to a large degree for weak NAIP5-NLRC4 flagellin sensing *in vivo* (Figs 1A, 3B and 5B). Together with the observation that TNF is important for AM but not neutrophil-mediated killing, these experiments highlight the importance of TNF-mediated antibacterial mechanisms in AM in the context of *L*. *pneumophila* lung infection.

Our results indicating the functionally relevant production of TNF by neutrophils and monocytes are in agreement with a study by Copenhaver et al. [67]. However, there it was found that AM and DCs are also important for TNF production in response to *L*. *pneumophila* lung infection. In contrast, we do not observe significant TNF production by AM, which may reflect differences in the strains of bacteria used between the studies. Though we also observed TNF production by DCs, our results with MN-TNF NAIP5^129S1^ mice suggest that neutrophil / monocyte-derived TNF is physiologically more relevant for the innate immune response to *L*. *pneumophila* (Fig 5A, [52]).

In light of the finding that neutrophil and monocyte-derived TNF mediates an essential AM-driven immune response that can compensate for weak NAIP5-NLRC4-mediated immunity, it is interesting to note that ΔFlaA *L*. *pneumophila* is able to replicate in AM within the first 2 days p.i., after which bacteria are cleared [23]. These kinetics fit with the observations that TNF peaks in the BALF 2 days p.i. [39,65], that macrophages require pre-activation of around 20 hours with TNF before they become restrictive for *L*. *pneumophila* replication (Fig 6A, [36]), and that failure to recruit neutrophils to the lung from 12 hours up to around 2 days p.i. does not greatly impact bacterial burden, though these kinetics may vary with the size of the inoculum [19,23]. In addition, anti-TNF Ab treatment of A/J mice resulted in an increase in lung bacterial burden only as of around day 3 p.i. [39]. Also consistent with a need for neutrophil-derived TNF is the observation that clearance of ΔFlaA *L*. *pneumophila* is delayed to 72 hours p.i. in IL1R^-/-^ mice, in which neutrophil recruitment is delayed, and that in MyD88^-/-^ mice clearance is postponed to 6 days p.i., or even abrogated [23]. Since MyD88^-/-^ BMDM fail to secrete TNF in response to *L*. *pneumophila* [36,68], and neutrophils secrete TNF in a flagellin-independent manner (Fig 3B, [10]), it seems highly likely that impaired TNF production by neutrophils and monocytes contributes to the striking susceptibility of MyD88^-/-^ mice to *L*. *pneumophila* lung infection. The fact that AM do not produce much TNF in response to *L*. *pneumophila* infection but instead rely mostly on neutrophils and monocytes, which must first be recruited to the airways to produce TNF, likely reflects a mechanism which limits overzealous lung inflammation. Indeed, TNF is a very potent cytokine, and it's leakage from the airspace to the circulation can on its own strongly contribute to anaphylactic shock, as shown by systemic anti-TNF treatment in a rabbit model of *Pseudomonas aeruginosa* pneumonia [69]. Congruent with this idea, though neutrophils are essential for the resolution of *L*. *pneumophila* lung infection, they are also associated with lung pathology in Legionnaires' disease [70,71]. This may in part be due to their role in TNF secretion.

We also show that neutrophils kill *L*. *pneumophila* in the lung directly by NOX2-dependent mechanisms. Interestingly, AM do not produce ROS in response to WT *L*. *pneumophila*. This is in line with a study demonstrating that *L*. *pneumophila* actively represses ROS in AM by a T4SS-dependent mechanism [54] and our observation that AM produce ROS in response to ΔT but not much ROS in response to WT *L*. *pneumophila* (Fig 3B). Why this mechanism is not active in neutrophils remains unclear, given that both neutrophils and AM are targeted by the T4SS and harbor live *L*. *pneumophila in vivo* (Fig 2A, [10]). In fact, for neutrophils the opposite is true, as our results show that ROS induction in neutrophils is T4SS-dependent. On a similar note, a recent study has shown differential responses between macrophages and neutrophils to *Salmonella* flagellin, in that NAIP5-NLRC4 triggered pyroptosis in macrophages but not neutrophils [72]. How *L*. *pneumophila* adapts to these two different intracellular environments also remains unknown. The differential activation of neutrophils and AM by *L*. *pneumophila* will likely yield interesting insights into this host-pathogen interaction in future investigations.

In this study, we show that the TNF-mediated antibacterial mechanism in AM is dependent on the rerouting of *L*. *pneumophila* to lysosomal compartments, where they are degraded via processes that involve acidification. This acidification likely occurs early in the infection cycle, since fusion of LCVs and lysosomes can be observed within an hour of infection in BMDM pre-treated with TNF. Consistent with this view, a previous study found that *L*. *pneumophila* has at least one T4SS effector, SidK, which inhibits the v-ATPase [73]. SidK is highly induced when *L*. *pneumophila* begins a new growth cycle, presumably counteracting the early acidification of LCVs [73]. The observation that bafilomycin A1 alone reduced *L*. *pneumophila* replication in BMDM is expected, since *L*. *pneumophila* requires the acidification of the LCV in late stages of infection for proper LCV maturation [74].

Our data implicate the involvement of at least one caspase other than caspase-1 or 11 in the TNF-mediated growth-restriction of *L*. *pneumophila* in macrophages, since the mechanism is active in caspase-1/11^-/-^ BMDM and can be partially blocked by Q-VD-OPh. Of the eight remaining caspases encoded in the mouse genome, namely caspase-2, 3, 6, 7, 8, 9, 12 and 14, a number have been shown to be involved in non-apoptotic functions related to host defense [75]. Caspase-7 has been shown to mediate the fusion of LCVs with lysosomes, though this was dependent on caspase-1 activity [42]. However, caspase-7 has also been demonstrated to protect cells from plasma membrane damage with the pore-forming toxin Listeriolysin O, and this was caspase-1 independent [76]. Caspase-8 has also been shown to mediate innate immune responses involving NFκB activation in response to dsRNA, as well as cell motility [77,78]. Caspases 7 and 8 might therefore be good candidates for involvement in the TNF-mediated mechanism.

Our results also implicate modest involvement of cathepsins or calpains in the TNF-mediated restriction of *L*. *pneumophila* replication in macrophages, as demonstrated by the partial inhibition of the TNF-mediated effect by E-64d. However, we did not find a requirement for cathepsin B, D, H, L or S, though a redundant requirement among these cathepsins cannot be excluded. Of note, the cathepsin B inhibitor CA-074-Me partially blocked the TNF-mediated restriction of *L*. *pneumophila* growth, however this was shown to be non-specific as the compound blocked the effect equally well in WT and CtsB^-/-^ BMDM (S3C Fig).

Further, we find that caspase and cathepsin or calpain activity may be interdependent. This may not be a surprising result, as cathepsins have been documented to have an involvement upstream of caspase activation in other biological contexts and *in vitro* [79,80]. Similarly, calpains have been shown to impact the activation of caspase-8, 9 and 12 [81–83]. Furthermore, the intracellular pathogen *Francisella tularensis* was shown to exploit this relationship to manipulate caspases and promote its survival in neutrophils [82]. Further investigation of these mechanisms will surely yield a better understanding of TNF-mediated host defense mechanisms directed at intracellular pathogens.

## Materials and Methods

### Ethics statement

This study was conducted in accordance to the guidelines of the animal experimentation law (SR 455.163; TVV) of the Swiss Federal Government. The protocol was approved by Cantonal Veterinary Office of the canton Zurich, Switzerland (Permit number 125/2012).

### Mice and *L*. *pneumophila* infections

All mice used in this study were bred at the Swiss Federal Institute of Technology Zürich or purchased (Janvier Labs, Le Genest Saint Isle, France) and used at 6–20 weeks of age (age- and sex-matched within experiments). All mice were backcrossed >9 generations on the C57BL/6 background with the exception of MN-TNF NAIP5^129S1^ mice. MemTNF KI mice and MN-TNF NAIP5^129S1^ mice have been previously described [52,84]. Sequencing of the MN-TNF NAIP5^129S1^ mice revealed the same mutations in 129S1 NAIP5 (NAIP5^129S1^) as previously described [49], with the exception of two mutations in exon 15, which matched the C57BL/6 DNA sequence. Bone marrow chimeric mice were generated as described previously [18], reconstituting with a total of 5 x 10^6^ bone marrow cells and allowing at least 8 weeks for reconstitution of lethally irradiated Ly5.1^+^ WT recipient mice. Neutrophil and AM chimerism was around 40:60 in WT:WT mice, 35:65 in WT:CYBB^-/-^ mice and 33:67 in WT:TNFR1^-/-^ mice.

The *L*. *pneumophila* strains used in this study were the wildtype strain JR32 (Philadelphia-1) [85], as well as modifications of JR32 including an aflagellated mutant (ΔFlaA) [86], JR32-GFP [87], JR32-GFPind (pGS-GFP-04) [88], a deletion mutant lacking a functional Icm/Dot T4SS (ΔT) [89], and ΔT-GFP [87]. *L*. *pneumophila* was grown for 3 days at 37°C on charcoal yeast extract (CYE) agar plates before use, with chloramphenicol (5 mg/ml) added for selection of strains containing GFP-encoding plasmids.

For intranasal (i.n.) infections mice were anesthetized with an i.p. injection of 5 mg xylazine/100 mg ketamine per gram body weight, and 5 x 10^6^ CFU *L*. *pneumophila* (unless otherwise specified) resuspended in 20 μl PBS were directly applied to one nostril using a Gilson pipette. Bacterial titers in bronchoalveolar lavage fluid (BALF) were determined by plating serial dilutions in PBS on CYE plates. For quantification of CFU from sorted AM and neutrophils, cells were lysed to release viable *L*. *pneumophila* by vortexing 30 seconds in 1 ml PBS with 0.7% Tween 20 prior to plating serial dilutions in PBS on CYE plates.

### *In vitro L*. *pneumophila* infection of BMDM

Bone marrow-derived macrophages (BMDM) were generated by plating bone marrow in L929 conditioned medium containing M-CSF in 5 cm diameter non-cell culture treated Petri dishes as described previously [18]. On day 7, BMDM were harvested in ice cold PBS, 5% FBS, 2.5 mM EDTA by incubating 12 min in the fridge and resuspending by pipetting. The cells were then seeded at 1 x 10^5^ cells/well in 96-well plates and rested overnight prior to infection. *L*. *pneumophila* used for infection was grown for 3 days at 37°C on CYE agar plates, then inoculated in ACES yeast extract medium at an OD600 of 0.1 and grown for 21 h at 37°C before use, with 5 mg/ml chloramphenicol added to maintain plasmids. BMDM were infected at MOI 0.1, synchronized by centrifugation, and incubated for 3 days at 37°C, 5% CO_2_. Intra- and extracellular CFU were quantified on day 3 by plating on CYE plates after a 10 min incubation in dH_2_O to lyse BMDM. Where indicated, 20 nM V-ATPase inhibitor bafilomycin A1 (Enzo Life Sciences, BML-CM110-0100), 25 μM cathepsin B inhibitor CA-074-Me (Enzo Life Sciences, BML-PI126-0001), 25 μM cathepsin D inhibitor pepstatin A (Enzo Life Sciences, ALX-260-085-M005), 2 μg/ml TNFR1-Fc (Adipogen, AG-40B-0074-C050), 25 μg/ml anti-IL1β (R&D, AB-401-NA), 25 μg/ml anti-TNF (Bioxcell, BE0058, clone XT3.11) or 100 ng/ml TNF (Peprotech, 315-01A) were added 15 min prior to infection.

### Microscopy experiments

BMDM were seeded in 24-well plates containing 0.01% polylysine solution (Sigma P4707) coated 12 mm cover glasses (Faust 6080181) at 2.5 x 10^5^ cells/well and rested overnight. Where indicated 100 ng/ml TNF (Peprotech, 315-01A) or 200 U/ml IFNγ was added to pre-activate the BMDM. Cells were infected with *Lpn*-GFP as described above at MOI 5 for 1 or 3 hours at 37°C, 5% CO_2_, with the simultaneous addition of100 ng/ml TNF or 200 U/ml IFNγ where indicated. For the final 30 minutes of incubation 1μM lysotracker Red DND-99 (Life Technologies, L7528) and 0.5 μg/ml Cholera toxin B AF647 (CTB-AF647, Life Technologies, C34778) were added to the cells. Cells were then washed with 1 ml PBS, and cover glasses were then placed on parafilm, and fixed 5–10 min at RT with 200 μl 4% PFA in PBS. Cells were washed 3 times with 200 μl PBS, incubating 2 min after applying each wash. Cover glasses were dipped in dH_2_O, blotted on paper towel to remove excess water and mounted on glass slides with cells facing downwards with 6 μl Mowiol (VWR, 475904–100). Z-stack images were acquired on a spinning-disk confocal microscope (Visitron confocal system) using a 100x objective, and analyzed with volocity software (PerkinElmer, Waltham, MA). To assess co-localization of *L*. *pneumophila* and lysosomes, at least 100 bacteria were scored per coverslip.

### Antibodies and flow cytometry

BALF was recovered from mice at the specified timepoint in 1 ml sterile PBS containing 5 mM EDTA as previously described [90]. Cells were surface stained 30 min in cold FACS buffer (PBS with 2.5% FBS, 5 mM EDTA) with Siglec-F (clone E50-2440, Biolegend), CD11c (clone N418, Biolegend), Ly6G (clone 1A8, BD Biosciences), Ly6C (clone AL-21, BD Biosciences, Allschwil, Switzerland), CD11b (clone M1/70, Biolegend), CD45.1 (clone A20, BD Biosciences), CD45.2 (clone 104, BD Biosciences).

For intracellular staining of TNF (clone MP6-XT22, Biolegend), mice were injected i.p. with 50 μl of 5 mg/ml Brefeldin A in EtOH (diluted with 100 μl PBS) 3 hours prior to taking BALF. Lavage was performed with 1 ml PBS 5mM EDTA containing 5 μg/ml Brefeldin A, and was immediately placed on ice. After surface stain, cells were washed with FACS buffer and fixed, permeabilized and stained using the BD Biosciences Cytofix/Cytoperm Kit according to the manufacturer's instructions. Data were acquired on an LSRII (BD Biosciences) and analyzed with FlowJo software (TreeStar, Ashland, OR). An Aria III instrument (BD Biosciences) was used for cell sorting.

### ROS assay

ROS was stained in BALF cells by collecting BALF as usual in 1 ml PBS 5 mM EDTA, washing with 2 ml RPMI 10% FBS at RT, and staining with 60 μM Dihydroethidium (Sigma, D7008) for 1 hour at 37°C, 5% CO_2_. For a positive control, cells were stimulated with PMA/ionomycin. Cells were then washed in 2 ml cold FACS buffer and stained as usual with fluorescence-labeled Abs. Data were acquired on an LSRII (BD Biosciences), Dihydroethidium was measured in the FITC channel.

### Statistical analysis

Non-parametric tests, including the Kruskal-Wallis test with Dunn's post test, the Mann-Whitney test, or in the case of paired samples, the Wilcoxon test, were applied for statistical analysis using Prism GraphPad software (La Jolla, CA).

## Supporting Information

S1 Fig TNF does not compromise BMDM viability. WT BMDM were seeded in 96 well plates at 1x10^5^ cells / well. After resting overnight, media was replaced with new media containing 20% L929 conditioned media containing M-CSF, with or without 100 ng/ml rTNF. After 3 days of incubation at 37°C, medium was replaced with 200 μl medium containing 20% L929 conditioned media and 10% alamar blue (Lucerna Chem AG, A1180), and incubated for 6.5 hr at 37°C. Conversion of alamar blue reagent by live cells was then measured with an ELISA plate reader and OD_570_-OD_600_ was calculated. Results are from one experiment.
(TIF)

S2 Fig BMDM secrete TNF in response to *L*. *pneumophila* infection. This secretion is downstream of MyD88 and independent of NLRC4. **(A)** WT, MN-TNF NAIP5^129S1^ or NLRC4^-/-^ BMDM were infected with WT *L*. *pneumophila* at MOI 0.1 or left untreated. **(A)** 3 days p.i. supernatant was collected and TNF was quantified by cytometric bead array assay.
(TIF)

S3 Fig TNF-mediated killing of *L*. *pneumophila* is partially blocked by CA-074-Me, via an off target effect. **(A)** Caspase-1/11^-/-^ BMDM were infected with WT *L*. *pneumophila* at MOI 0.1. Where indicated rTNF and/or cathepsin B inhibitor CA-074-Me were added at the time of infection. 3 days p.i. BMDM were lysed and CFU were quantified on CYE agar plates. Data are from 2 pooled experiments. **(B)** WT or TNFR1^-/-^ BMDM were either pre-treated with rTNF overnight or left untreated, and then infected with ΔFlaA *L*. *pneumophila* at MOI 0.1. Where indicated rTNF and/or cathepsin B inhibitor CA-074-Me were added at the time of infection. 3 days p.i. BMDM were lysed and CFU were quantified on CYE agar plates. Data are from 2 pooled experiments. **(C)** WT or CtsB^-/-^ BMDM were either pre-treated with rTNF overnight or left untreated, and then infected with ΔFlaA *L*. *pneumophila* at MOI 0.1. Where indicated rTNF and/or cathepsin B inhibitor CA-074-Me was added at the time of infection. 3 days p.i. BMDM were lysed and CFU were quantified on CYE agar plates. Data are from 2 pooled experiments. **p<0.01 by Mann-Whitney test.**¨**
(TIF)

S4 Fig TNF-mediated killing of *L*. *pneumophila* is partially blocked by Z-VAD-FMK and Q-VD-OPh. WT BMDM were either pre-treated with rTNF overnight or left untreated, and then infected with ΔFlaA *L*. *pneumophila* at MOI 0.1. Where indicated rTNF and/or Z-VAD-FMK or Q-VD-OPh were added at the time of infection. 3 days p.i. BMDM were lysed and CFU were quantified on CYE agar plates. Data is from 1 experiment.
(TIF)
