# Supplementary figures and images for: Neutrophil and Alveolar Macrophage-Mediated Innate Immune Control of Legionella pneumophila Lung Infection via TNF and ROS
Source: PLoS Pathog. 2016 Apr 22;12(4):e1005591. doi: 10.1371/journal.ppat.1005591 (PMC4841525; doi:10.1371/journal.ppat.1005591)

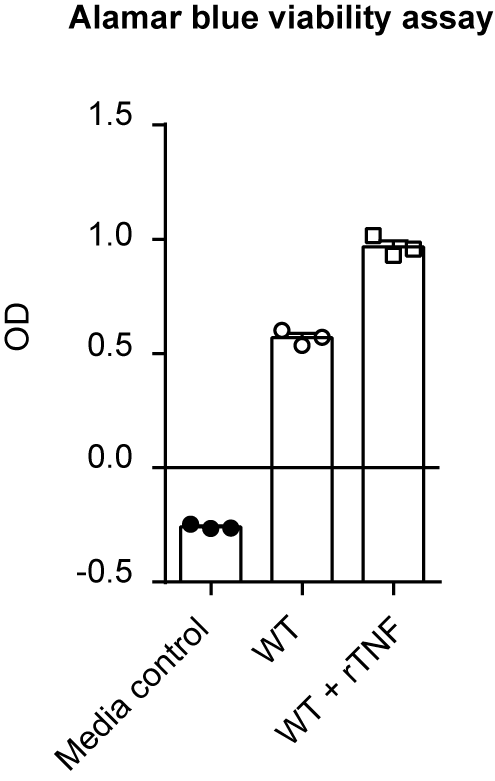

Supplement: S1 Fig — WT BMDM were seeded in 96 well plates at 1x10^5^ cells / well. After resting overnight, media was replaced with new media containing 20% L929 conditioned media containing M-CSF, with or without 100 ng/ml rTNF. After 3 days of incubation at 37°C, medium was replaced with 200 μl medium containing 20% L929 conditioned media and 10% alamar blue (Lucerna Chem AG, A1180), and incubated for 6.5 hr at 37°C. Conversion of alamar blue reagent by live cells was then measured with an ELISA plate reader and OD570-OD600 was calculated. Results are from one experiment. (TIF) [file ppat.1005591.s001.tif]

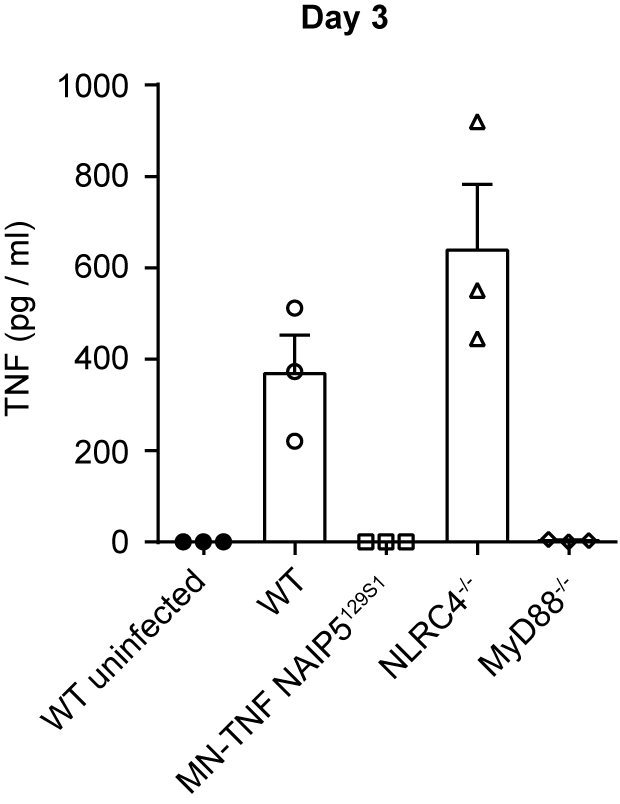

Supplement: S2 Fig — (A) WT, MN-TNF NAIP5129S1 or NLRC4-/- BMDM were infected with WT L. pneumophila at MOI 0.1 or left untreated. (A) 3 days p.i. supernatant was collected and TNF was quantified by cytometric bead array assay. (TIF) [file ppat.1005591.s002.tif]

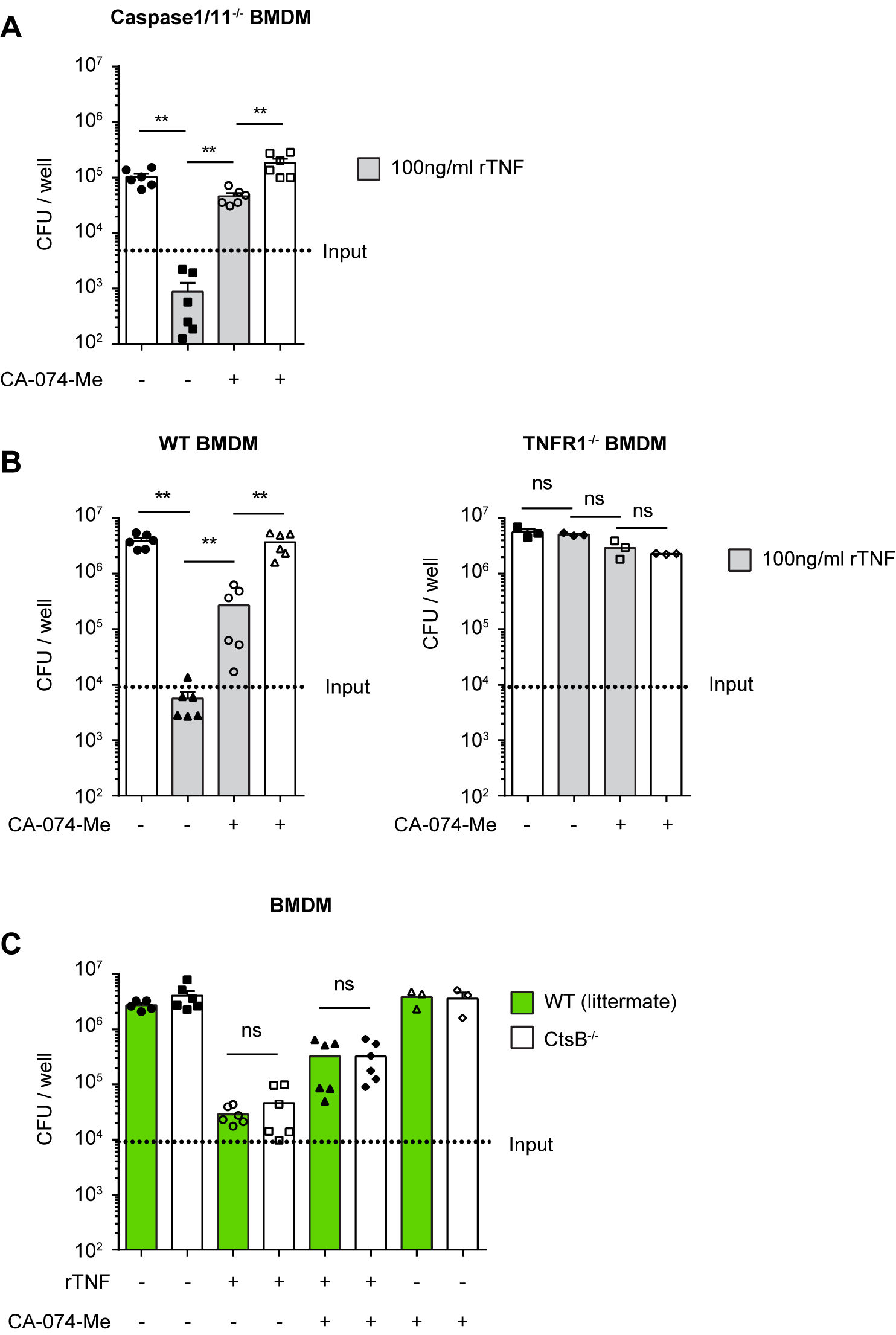

Supplement: S3 Fig — (A) Caspase-1/11-/- BMDM were infected with WT L. pneumophila at MOI 0.1. Where indicated rTNF and/or cathepsin B inhibitor CA-074-Me were added at the time of infection. 3 days p.i. BMDM were lysed and CFU were quantified on CYE agar plates. Data are from 2 pooled experiments. (B) WT or TNFR1-/- BMDM were either pre-treated with rTNF overnight or left untreated, and then infected with ΔFlaA L. pneumophila at MOI 0.1. Where indicated rTNF and/or cathepsin B inhibitor CA-074-Me were added at the time of infection. 3 days p.i. BMDM were lysed and CFU were quantified on CYE agar plates. Data are from 2 pooled experiments. (C) WT or CtsB-/- BMDM were either pre-treated with rTNF overnight or left untreated, and then infected with ΔFlaA L. pneumophila at MOI 0.1. Where indicated rTNF and/or cathepsin B inhibitor CA-074-Me was added at the time of infection. 3 days p.i. BMDM were lysed and CFU were quantified on CYE agar plates. Data are from 2 pooled experiments. **p<0.01 by Mann-Whitney test.¨ (TIF) [file ppat.1005591.s003.tif]

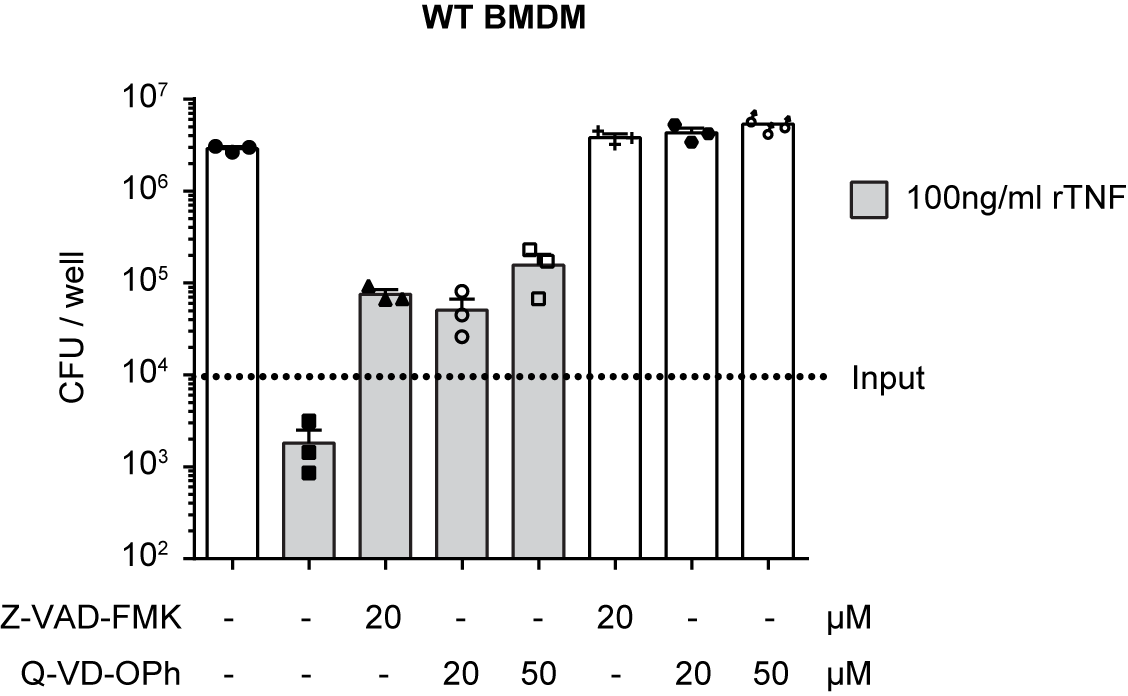

Supplement: S4 Fig — WT BMDM were either pre-treated with rTNF overnight or left untreated, and then infected with ΔFlaA L. pneumophila at MOI 0.1. Where indicated rTNF and/or Z-VAD-FMK or Q-VD-OPh were added at the time of infection. 3 days p.i. BMDM were lysed and CFU were quantified on CYE agar plates. Data is from 1 experiment. (TIF) [file ppat.1005591.s004.tif]
